# Supplementary material for: Apical contacts stemming from incomplete delamination guide progenitor cell allocation through a dragging mechanism
Source: eLife. 2021 Aug 27;10:e66483. doi: 10.7554/eLife.66483 (PMC8460252; doi:10.7554/eLife.66483)
Supplement: Transparent reporting form [file elife-66483-transrepform1.docx]

***eLife’s* transparent reporting form**

We encourage authors to provide detailed information *within their submission* to facilitate the interpretation and replication of experiments. Authors can upload supporting documentation to indicate the use of appropriate reporting guidelines for health-related research (see [EQUATOR Network](http://www.equator-network.org/%20)), life science research (see the [BioSharing Information Resource](https://biosharing.org/)), or the [ARRIVE guidelines](http://www.plosbiology.org/article/info:doi/10.1371/journal.pbio.1000412) for reporting work involving animal research. Where applicable, authors should refer to any relevant reporting standards documents in this form.

If you have any questions, please consult our Journal Policies and/or contact us: [editorial@elifesciences.org](mailto:editorial@elifesciences.org).

**Sample-size estimation**

- You should state whether an appropriate sample size was computed when the study was being designed
- You should state the statistical method of sample size computation and any required assumptions
- If no explicit power analysis was used, you should describe how you decided what sample (replicate) size (number) to use

Please outline where this information can be found within the submission (e.g., sections or figure legends), or explain why this information doesn’t apply to your submission:

The information about the sample size can be found in the legend of each figure. Each experiment was performed in a minimum number of 3 embryos. There are also measurements involving a single embryo giving the variations between embryos, which involved the comparison of cells (or cellular structures) within the embryo, and in these cases the sample size was often close to the size of the entire cell population.

**Replicates**

- You should report how often each experiment was performed
- You should include a definition of biological versus technical replication
- The data obtained should be provided and sufficient information should be provided to indicate the number of independent biological and/or technical replicates
- If you encountered any outliers, you should describe how these were handled
- Criteria for exclusion/inclusion of data should be clearly stated
- High-throughput sequence data should be uploaded before submission, with a private link for reviewers provided (these are available from both GEO and ArrayExpress)

Please outline where this information can be found within the submission (e.g., sections or figure legends), or explain why this information doesn’t apply to your submission:

All experiments were performed at least three times which correspond to technical replication. Biological replication depends of the experiments, it corresponds to embryos or cells, each biological replication is indicated in the legends of figures.

**Statistical reporting**

- Statistical analysis methods should be described and justified
- Raw data should be presented in figures whenever informative to do so (typically when N per group is less than 10)
- For each experiment, you should identify the statistical tests used, exact values of N, definitions of center, methods of multiple test correction, and dispersion and precision measures (e.g., mean, median, SD, SEM, confidence intervals; and, for the major substantive results, a measure of effect size (e.g., Pearson's r, Cohen's d)
- Report exact p-values wherever possible alongside the summary statistics and 95% confidence intervals. These should be reported for all key questions and not only when the p-value is less than 0.05.

Please outline where this information can be found within the submission (e.g., sections or figure legends), or explain why this information doesn’t apply to your submission:

Unless indicated, each plot displays the mean and the standard deviation. Statistical inference analysis was conducted initially by a Shapiro-Wilk test to assess if the data were normally distributed. Two-sample F-test of equality of variance was applied to assess if the samples had the same variance. Significance for two groups with normal distribution was calculated through a two-tail t-test and the p-value was selected depending if the dataset had or not equality of variance. For other distributions, a non-parametric Kolmogorov-Smirnov test or Mann-Whitney test was applied. The p-values indicated in the main figures were assigned as follow: ns: *p* > 0.05, *: *p* ≤ 0.05, **: *p* ≤ 0.01 and ***: *p* ≤ 0.001. All descriptive and inferential statistics can be found in source data files linked to the figures.

(For large datasets, or papers with a very large number of statistical tests, you may upload a single table file with tests, Ns, etc., with reference to sections in the manuscript.)

**Group allocation**

- Indicate how samples were allocated into experimental groups (in the case of clinical studies, please specify allocation to treatment method); if randomization was used, please also state if restricted randomization was applied
- Indicate if masking was used during group allocation, data collection and/or data analysis

Please outline where this information can be found within the submission (e.g., sections or figure legends), or explain why this information doesn’t apply to your submission:

To compare DFCs and EVL cells, cells were identified by position and morphological aspects.

To differentiate attached DFCs from detached DFCs, cells were identified by the presence (attached) or absence (detached) of actomyosin enrichment at the apical face.

For morpholino experiments, Tg(*sox17::utrn-GFP*) embryos at 1-cell stage were separated into randomized groups. One of the groups was injected with e-cad-MO plus phenol red and other group (control) was injected with phenol red in distillated water.

To analyze DFC protrusion interactions, embryos carrying at least two clusters of DFCs were selected by visual inspection.

All this information can be found in the Material and Methods or in the main text sections.

**Additional data files (“source data”)**

- We encourage you to upload relevant additional data files, such as numerical data that are represented as a graph in a figure, or as a summary table
- Where provided, these should be in the most useful format, and they can be uploaded as “Source data” files linked to a main figure or table
- Include model definition files including the full list of parameters used
- Include code used for data analysis (e.g., R, MatLab)
- Avoid stating that data files are “available upon request”

Please indicate the figures or tables for which source data files have been provided:

All numerical raw data together with descriptive and inferential statistics can be found as source data files linked to Figures 1 to 8.
